# Comparison of Biotinylated Monoclonal and Polyclonal Antibodies in an Evaluation of a Direct Rapid Immunohistochemical Test for the Routine Diagnosis of Rabies in Southern Africa

**DOI:** 10.1371/journal.pntd.0003189

**Published:** 2014-09-25

**Authors:** Andre Coetzer, Claude T. Sabeta, Wanda Markotter, Charles E. Rupprecht, Louis H. Nel

**Affiliations:** 1 Department of Microbiology and Plant Pathology, University of Pretoria, Gauteng, South Africa; 2 Agricultural Research Council-Onderstepoort Veterinary Institute, Rabies Division, Gauteng, South Africa; 3 Ross University School of Veterinary Medicine, Basseterre, St. Kitts, West Indies; Swiss Tropical and Public Health Institute, Switzerland

## Abstract

The major etiological agent of rabies, rabies virus (RABV), accounts for tens of thousands of human deaths per annum. The majority of these deaths are associated with rabies cycles in dogs in resource-limited countries of Africa and Asia. Although routine rabies diagnosis plays an integral role in disease surveillance and management, the application of the currently recommended direct fluorescent antibody (DFA) test in countries on the African and Asian continents remains quite limited. A novel diagnostic assay, the direct rapid immunohistochemical test (dRIT), has been reported to have a diagnostic sensitivity and specificity equal to that of the DFA test while offering advantages in cost, time and interpretation. Prior studies used the dRIT utilized monoclonal antibody (MAb) cocktails. The objective of this study was to test the hypothesis that a biotinylated polyclonal antibody (PAb) preparation, applied in the dRIT protocol, would yield equal or improved results compared to the use of dRIT with MAbs. We also wanted to compare the PAb dRIT with the DFA test, utilizing the same PAb preparation with a fluorescent label. The PAb dRIT had a diagnostic sensitivity and specificity of 100%, which was shown to be marginally higher than the diagnostic efficacy observed for the PAb DFA test. The classical dRIT, relying on two-biotinylated MAbs, was applied to the same panel of samples and a reduced diagnostic sensitivity (83.50% and 90.78% respectively) was observed. Antigenic typing of the false negative samples indicated all of these to be mongoose RABV variants. Our results provided evidence that a dRIT with alternative antibody preparations, conjugated to a biotin moiety, has a diagnostic efficacy equal to that of a DFA relying on the same antibody and that the antibody preparation should be optimized for virus variants specific to the geographical area of focus.

## Author Summary

Rabies is a neglected disease that primarily affects poor rural communities of the developing world. Lack of surveillance, related to limited diagnostic capabilities, contributes to the underestimation of the burden of this disease. Here we report an evaluation of the direct immunohistochemical test (dRIT) as a method for routine rabies diagnosis in southern Africa. The dRIT has potential as a practical and cost-effective test that may improve rabies diagnostic capacities where it is most needed, and with this work we hope to contribute to the advancement of the dRIT as a more generally accepted and applied method. For the first time, we have evaluated a modification of the dRIT in which a polyclonal antibody preparation was biotinylated and compared to the monoclonal antibodies used for the development of all subsequent experimental applications of the dRIT to date. We conclude that the dRIT is a superior test for rabies diagnosis that is easily adaptable to tolerate the use of different antibody preparations. We further demonstrate that the assay should be optimized with respect to the virus variants of the region where it is to be implemented.

## Introduction

Rabies is a neglected zoonosis that is responsible for the death of tens of thousands of people per annum [1]. The majority of human rabies deaths are associated with canine rabies in resource-limited countries. Rabies is caused by multiple lyssaviruses (Genus: *Lyssavirus*, Family: *Rhabdoviridae*), of which the prototype is rabies virus (RABV). While RABV is most important from a global disease perspective, there are more than 12 other lyssavirus species, most of which have been associated with infrequent cases of human rabies [2], [3]. Although classical rabies has the highest known case-fatality rate of any infectious disease, and is preventable by means of effective pre- and post-exposure prophylaxis, the disease is still widespread throughout developing countries on the African and Asian continents [1], [4]–[7]. The process of post-mortem diagnostic confirmation of rabies plays a crucial role in general disease surveillance and is also involved in disease management programs for animal populations (e.g. identifying disease outbreaks within geographical regions where dog vaccination campaigns are being implemented), as well as in risk assessments for consideration of human prophylaxis.

In the case of resource-limited developing countries, where limited or no diagnostic confirmation is undertaken, very little rabies data are reported to relevant authorities. In some instances it has also been found that even though limited diagnosis may occur, the diagnostic results are not reported to the relevant authorities at all. This appears to be due to various logistical reasons, such as a lack of record keeping, limited communication, etc. [8]. As a result of the under estimation of the disease in animal populations, developing countries typically give little or no support and rabies remains of low political priority [1], [8]. The demonstration of the disease burden is thus dependent on proper surveillance and diagnostic activities to break this cycle of neglect.

The gold standard assay for rabies diagnosis is the direct fluorescent antibody (DFA) test [9], [10], but proper application of this method in much of the developing world remains limited. This is due in part to a lack of: i) stable infrastructure -power supply, easy access to running water and good quality waste disposal; ii) preservation of cold chains during sample transport; iii) well equipped diagnostic laboratories; and iv) a quality management system [11]. The development of diagnostic assays that are more suitable for routine application in developing countries has undergone major advances, with the innovation of numerous novel diagnostic assays [12]–[14]. Among the rabies diagnostic assays that are potentially advantageous for low-resource settings, the direct rapid immunohistochemical test (dRIT) has, to date, shown promise in preliminary applications. This test has been shown to have a diagnostic sensitivity and specificity equal to that of the DFA test, but requires smaller initial capital investment and may offer other significant advantages [15]. For example, the dRIT can be performed on fresh, frozen or glycerol-preserved samples using basic equipment such as a light microscope, that does not need an external power supply. The dRIT can also be performed in a shorter time than required routinely for the DFA and decentralized implementation of such a method may be helpful in overcoming crucial lack of sample submission, due to poor infrastructure and cost of transport [14]–[19].

To date, studies on the dRIT method have utilized monoclonal antibody (MAb) preparations (“anti-N 502”, etc. [20]) [14], [16]–[19]. The objective of this study was to test the hypothesis that an alternative polyclonal antibody (PAb) preparation could be biotinylated and applied in the dRIT diagnostic reaction with equal or improved results compared to the use of the MAbs. The use of alternative antibody preparations could also contribute to a more widespread application of the dRIT.

Our approach was to use a PAb preparation, which is used routinely for the DFA test (after fluorescein isothiocyanate labelling) in South Africa and a number of southern African countries [21]. A dRIT assay using this biotinylated PAb was compared with the dRIT using reference MAb 1 and MAb 2 as used in other studies, and the DFA (using the PAb). In our hands the PAb dRIT was marginally more accurate than the DFA assay using the same PAb preparation. For this cohort of African viruses, the PAb preparation improved the diagnoses of some mongoose rabies virus antigens, in comparison to the dRIT assays in which two MAbs were used.

## Methods

### Biotinylated antibodies used in the study

#### Biotinylation of the anti-ribonucleoprotein polyclonal antibody preparation

The PAb preparation used in this study was produced at the Agricultural Research Council-Onderstepoort Veterinary Institute (ARC-OVI), Rabies Division, by immunizing goats with purified ribonucleoprotein (RNP) antigens obtained from two lyssavirus species (a RABV laboratory strain SAG-2 and Mokola virus (MOKV, 229/97)) according to the standard operating procedure [22]. The unlabelled PAb preparation was biotinylated using an EZ-Link Sulfo-NHS-Biotinylation Kit (Thermo Scientific) according to the manufacturer's instructions. Briefly, the biotinylation process was performed by mixing 10 mg/ml of the clarified PAb preparation with 268 µl reconstituted Sulfo-NHS biotin compound (10 mM, sulfosuccinimidyl-6-[biotin-amido]-hexanoate). The reaction mixture was incubated on ice for two hours and then desalted with a Zeba desalt spin column (Thermo Scientific). Subsequent to the antibody biotinylation, the quantification of the biotinylation was determined using a HABA/Avidin assay (Thermo Scientific). The optical density values, molecular weight and concentration of the PAb preparation was applied in the “HABA calculator” (http://www.piercenet.com/haba/) to determine the molar ratio of biotin to the polyclonal antibody.

#### Biotinylated anti-nucleocapsid monoclonal antibodies

To date, biotinylated MAbs have been used routinely as a cocktail of highly concentrated antibodies. During this study, however, the diagnostic efficacy of two individual MAbs was investigated, as previously described [16]. The MAbs, binding to two different epitopes on the nucleoprotein, were supplied as two individual ready-to-use vials (MAb 1 and MAb 2).

### Sample size and selection

#### Ethics statement

Animal rabies is a notifiable disease in South Africa. State veterinarians had submitted central nervous system (CNS) tissue samples to the OIE Rabies Reference Laboratory, the ARC-OVI, Rabies Division, for routine rabies diagnosis based on suspicion of rabies. As part of its mandate, the Reference Laboratory performs routine rabies diagnosis for disease control and management of bite victims on behalf of the Department of Agriculture Fisheries and Forestry (DAFF).

All the CNS samples for the rabies-related viruses used in this study were from virus stocks that had been prepared according to the diagnostic procedures of the Rabies Laboratory at the ARC-OVI, Rabies Division (ARC-OVI Ethical approval, 15/4P001). The other samples used in this study were selected from a much larger set of archived samples submitted to the ARC-OVI, Rabies Division, for routine rabies diagnosis over a period of two years (year: 2011–2012), while a sub-set of 30 archival samples (year: 1999) were included. This study thus only relied on archived CNS tissues and did not require specific ethical clearance as no live animal models were used but permission was granted by the Director of the Institute.

“The animal experimental protocols, animal caging and care, as well as end point for the animal experiments performed at the ARC-OVI, Rabies Division, were approved by the Animal Ethics Committee for the use of living vertebrates for research, diagnostic procedures and product development (Agricultural Research Council-Onderstepoort Veterinary Institute, South Africa) under 15/4/P001.”

#### Sample cohort

The sample set used in this study consisted of 255 central nervous system (CNS) samples. Of the 255 samples, 249 were derived from the following mammalian species: domestic dog (*Canis familiaris*; n = 132), domestic cat (*Felis domesticus*; n = 27), black-backed jackal (*Canis mesomelas*; n = 26), bat-eared fox (*Otocyon megalotis*; n = 11), yellow mongoose (*Cynictis penicillata*; n = 26) and cattle (*Bos taurus*; n = 27) (Table S1). The species chosen for this study were selected based on their importance as reservoirs, maintenance hosts, or indicator species for rabies virus infection in southern Africa [23].

The remaining CNS tissue samples (n = 6) had been derived from mouse models, each infected with one of six southern African representative lyssavirus isolates (Table S2). The southern African lyssavirus isolates were proliferated in 2–3 day old suckling mice (ARC-OVI Ethical approval, 15/4P001) [24].

Prior to performing diagnosis on the chosen samples, each of the CNS samples was placed in a sterile petri dish and small pieces of tissue were removed from multiple sites to ensure that viral antigens were obtained from representative segments of the CNS sample. The composite samples were homogenized using a mortar and pestle to facilitate an efficient supply of viral antigens throughout each of the investigated samples.

### Direct fluorescent antibody test

**Figure 1:**
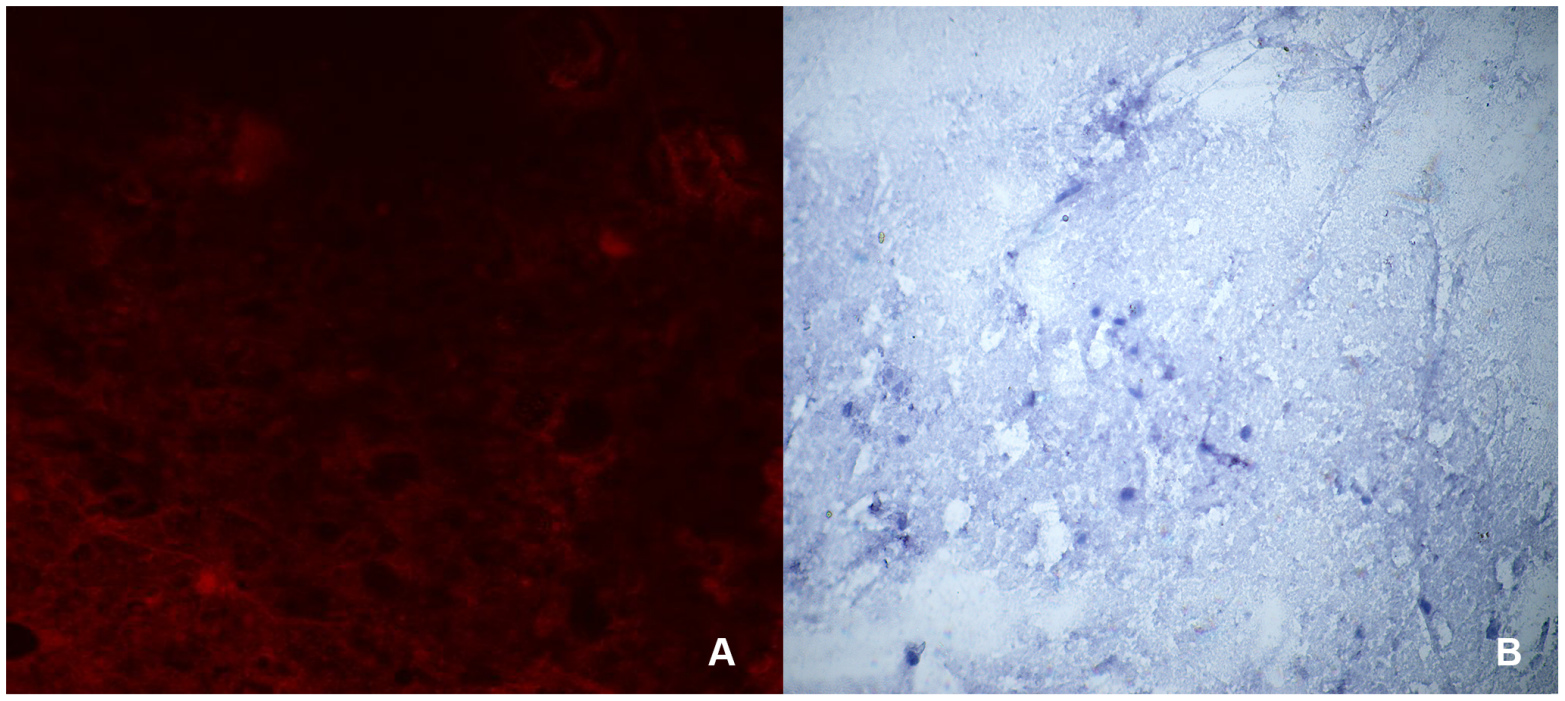
Touch impression of a rabies-negative domestic dog brain tested with the direct fluorescent antibody test (A) and direct rapid immunohistochemical test (B). (A) No immunofluorescence observed in the brain processed by DFA. Magnification, ×400. (B) No magenta inclusions are visible on the blue neuronal background of the brain processed by dRIT. Magnification, ×200.

**Figure 2:**
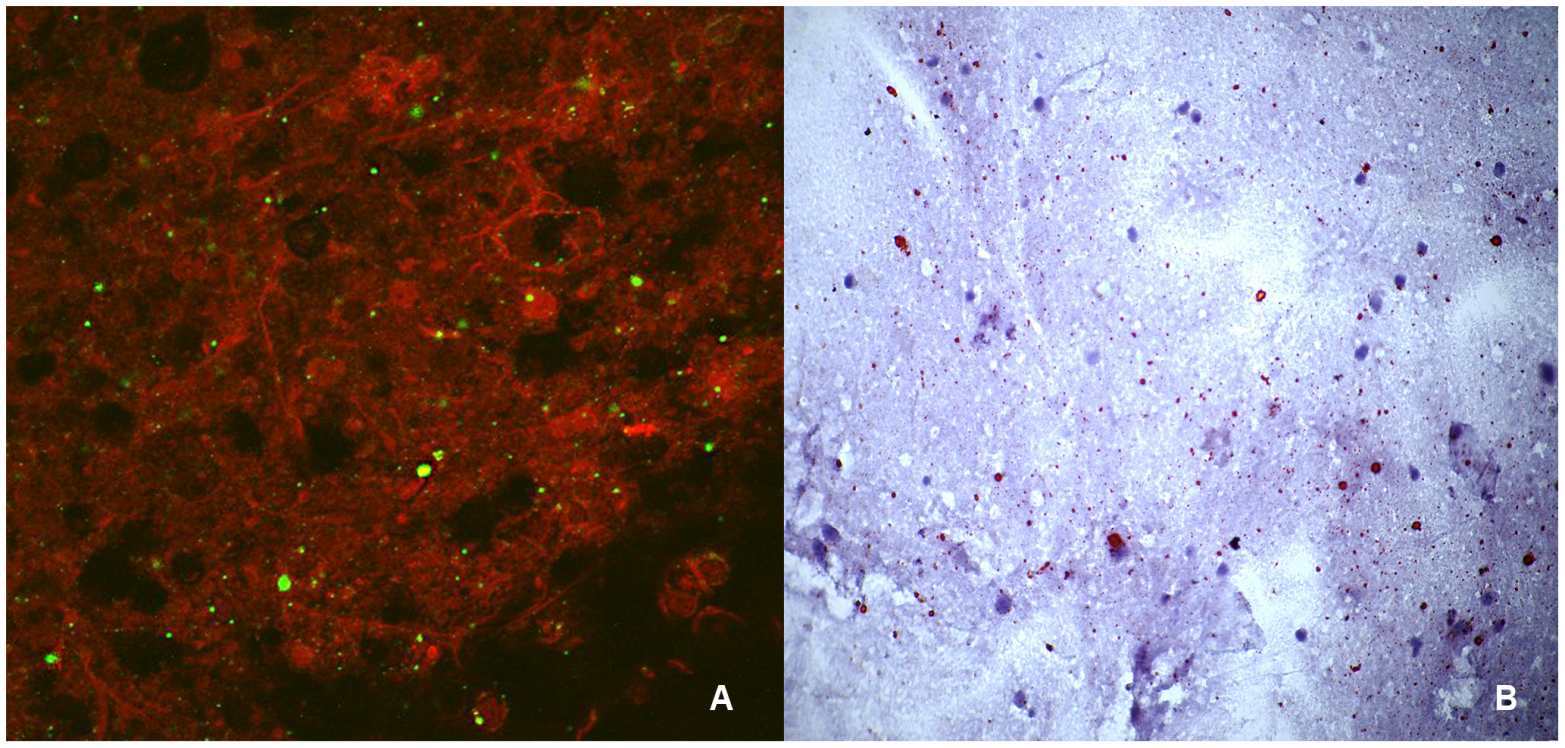
Touch impression of a rabies-positive domestic dog brain tested with the direct fluorescent antibody test (A) and direct rapid immunohistochemical test (B). (A) Apple-green immunofluorescent viral inclusions observed on the red neuronal tissue in the brain processed by DFA. Magnification, ×400. (B) Magenta viral inclusions are visible on the blue neuronal background of the brain processed by dRIT. Magnification, ×200.

All the samples included in this study (n = 255) were tested initially with the DFA test to determine the immunoreactivity scores associated with each of the samples [25]. The DFA test, relying on the FITC-labelled anti-ribonucleoprotein PAb preparation (ARC-OVI, Rabies Division) was performed according to the standard operating procedure [10]. To improve the contrast of the image and to reduce the levels of observed background staining, all tissue impressions were counterstained with an Evans blue counterstain (0.5% in PBS) before interpreting the final immunoreactivity. Negative results were based on a lack of apple-green fluorescing inclusion bodies (Figure 1A), while rabies-positive results were based on the presence of apple-green fluorescent inclusions visible within reddish counterstained neuronal tissue (Figure 2A).

### Direct, rapid immunohistochemical test

The dRIT diagnostic assay was performed in triplicate on all the CNS samples according to the published standard operating procedure using one of the three-biotinylated antibodies (MAb 1, MAb 2 and PAb), as described [16]. The results of the dRIT were produced while the DFA result was not known to the operator at the time when the dRIT results were interpreted. Negative results were based on a lack of magenta inclusion bodies on the blue neuronal background (Figure 1B), while positive results were based on the presence of magenta inclusions visible on a blue neuronal background (Figure 2B). For any samples that produced a different result with any of the tests, all the tests were repeated a further three times to ensure that all tests were performed correctly.

### Molecular investigation of false-positive results

#### Hemi-nested polymerase chain reaction

The total RNA extraction of any false RABV-positive CNS samples was performed using the Trizol reagent (Sigma-Aldrich) according to the manufacturer's instructions and the subsequent viral cDNA synthesis was performed according to a protocol published previously [26]. The subsequent hemi-nested-PCR (hn-PCR) amplification of the RABV nucleoprotein gene was performed according to a published protocol [26]–[28]. The hn-PCR product was extracted from the agarose gel and purified using the Wizard SV Gel and PCR clean-up system (Promega) according to the manufacturer's instructions.

#### Sequencing and phylogenetic analysis of the purified hn-PCR product

Both the forward and reverse strands of the purified PCR amplicon were sequenced using the hn-PCR primers and the BigDye Terminator v3.1 sequencing reaction kit according to the manufacturer's instructions (Applied Biosystems). The sequencing reactions were precipitated according to the manufacturer's instructions (Applied Biosystems) and subsequently sequenced using an ABI 3100 automated capillary sequencer (Applied Biosystems, University of Pretoria). The sequences obtained from both the forward and reverse primers were used to create a trimmed consensus sequence of 466 nt using CLC Main Workbench (CLC bio, Version 7.0) and subsequently subjected to a BLAST analysis on the GenBank website.

After the assembly of the consensus sequence, an alignment was created using the ClustalW subroutine of the BioEdit software [29]. A Maximum likelihood phylogenetic analysis was subsequently performed using the “Kimura-2” parameter (determined by JModel test software, Version 2.1.3) in MEGA (Version 2.10), with an estimated bootstrap support of 1000 replicates.

### Antigenic and molecular investigation of false-negative results

All RABV-positive tissue samples that produced apparent false-negative dRIT results with any of the biotinylated antibody preparations were subjected to antigenic typing and a molecular analysis to investigate the origin of the discrepancies in the observed immunoreactivity.

#### Antigenic typing of false-negative results

All the aforementioned samples were typed as either a canid or mongoose RABV variant using a panel of sixteen MAbs supplied by the Centre of Expertise for Rabies (Canadian Food Inspection Agency, Ottawa, Canada) [30], [31].

#### Quantifying viral RNA using real-time PCR amplification

The total RNA extraction was performed using the Trizol reagent (Sigma-Aldrich) according to the manufacturer's instructions, and an established “one-step” quantifying real-time PCR assay [28] was performed to quantify the amount of viral RNA present in all the samples.

### Data analysis

The determination of the diagnostic sensitivity, specificity, Cohen's kappa value and respective confidence intervals of the dRIT diagnostic assays was determined using an exact binomial distribution (MedCalc 12.2.1.0, Ostend Belgium).

## Results

### Immunoreactivity scores associated with both the DFA and dRIT diagnostic assays

Of the 255 samples tested in the study, the PAb dRIT diagnostic assay produced one false-positive result in comparison to the DFA test. In contrast, the dRIT diagnostic reaction, relying on either of the two-biotinylated MAbs (MAb 1 and MAb 2), apparently produced several incorrect results. The dRIT diagnostic assay relying on MAb 1 produced 34 false-negative results and a single false-positive result, while the dRIT diagnostic assay relying on MAb 2 produced 19 false-negative results and two false-positive results.

### Investigation of false-positive results

Two samples that were characterised as lyssavirus-negative according to the DFA test, tested positive with the dRIT diagnostic assay. The first sample (sample: 664/12; Table S1) was collected from a dog in the Limpopo province of South Africa, and produced a false-positive result with the dRIT diagnostic assay with MAb 2. The second sample (sample: 711/12; Table S1) was collected from a dog in the Mpumalanga province of South Africa, and produced a false-positive result with the dRIT diagnostic assay using any of the three-biotinylated antibodies (MAb 1, MAb 2 or PAb).

**Figure 3:**
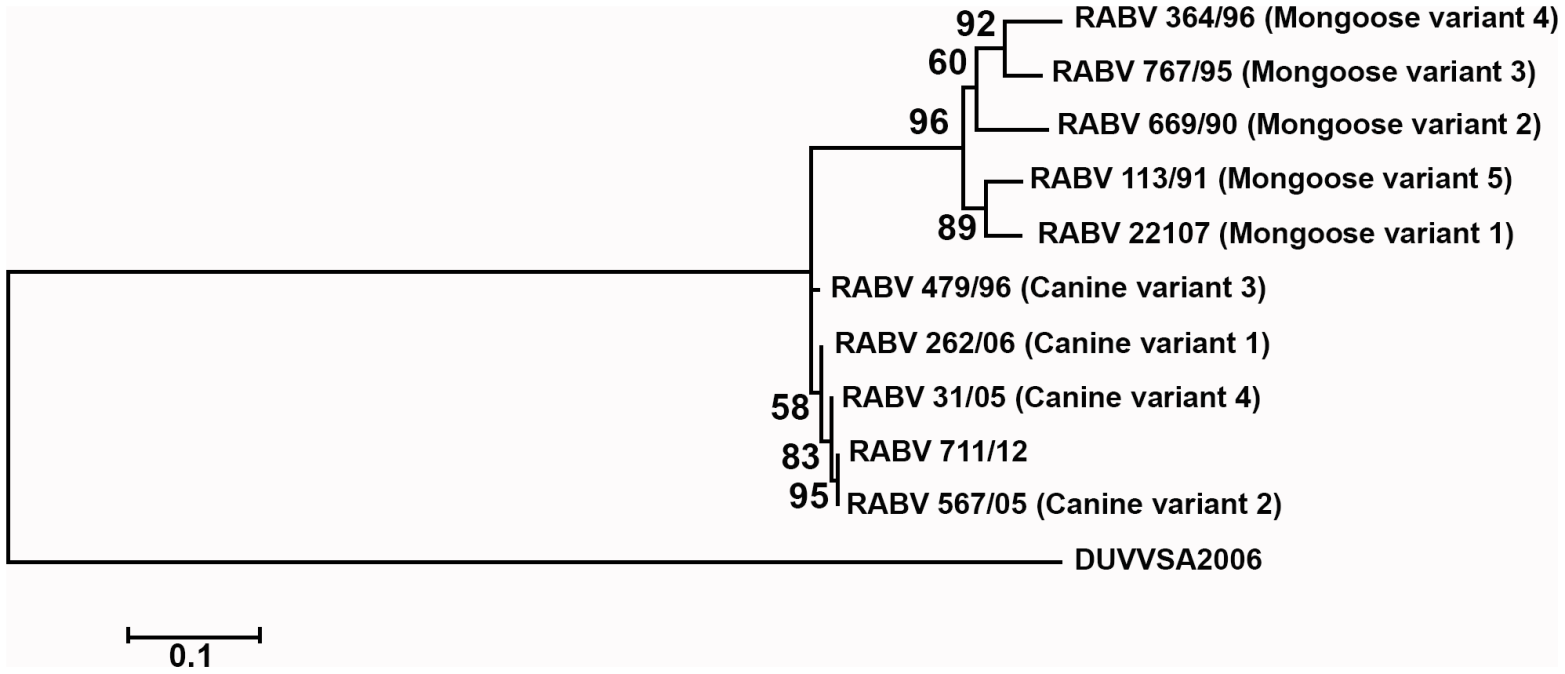
Phylogenetic representation of the genetic relationship between the rabies virus-positive sample (711/12) and representative canine and mongoose rabies virus variants circulating in southern Africa.

After applying the hn-PCR amplification, sample 711/12 contained viral nucleic acid that was amplified and used for further analysis. The BLAST analysis of the trimmed consensus sequence indicated that the amplified nucleic acid had a maximum identity of 99% with the nucleoprotein sequence of the RABV 567/04 isolate (GenBank Accession number: HM179505). The RABV 567/04 sequence belonged to the canid RABV variant, which was isolated from the same province of South Africa (Mpumalanga province) in 2004 [32]. The fact that the sample contained a canid variant of the RABV was further supported by phylogeny, which differentiated the mongoose and canid RABV variants (Figure 3). The molecular and phylogenetic evidence obtained here indicated that the DFA had missed a lyssavirus-positive sample (sample: 711/12), which was subsequently confirmed by dRIT.

### Investigation of false-negative results

From the sample cohort, 36 samples (*Canis familiaris*, n = 4; *Felis domesticus*, n = 14; *Canis mesomelas*, n = 1; *Cynictis penicillata*, n = 14; *Bos taurus*, n = 3) gave false-negative results with either one or both of the dRIT assays using the MAbs (Table 1). To further investigate, we performed antigenic typing and molecular characterisation of all these samples, and also included those mongoose samples that tested positive (n = 7) with both MAbs (Table 1).

**Table 1:** Summary of the viral RNA concentration and antigenic variants of the rabies-positive neuronal tissue samples that produced false-negative results subsequent to the application of the direct rapid immunohistochemical test using either one or both of the biotinylated monoclonal antibody preparations (MAb 1 or MAb 2).

Reference number	Host species#	Rabies virus variant	Viral RNA copy number per ng total isolated RNA
CNS samples delivering false-negative results with Monoclonal antibody #1			
756/99	Canid	Mongoose variant	5.49×10^2^
601/99	Feline	Mongoose variant	1.01×10^7^
620/99	Feline	Mongoose variant	1.89×10^4^
114/11	Feline	Mongoose variant	4.65×10^4^
376/11	Feline	Mongoose variant	3.08×10^2^
660/11	Feline	Mongoose variant	9.27×10^4^
261/12	Feline	Mongoose variant	1.63×10^5^
382/12	Feline	Mongoose variant	5.28×10^6^
540/99	Yellow mongoose	Mongoose variant	1.68×10^7^
1087/99	Yellow mongoose	Mongoose variant	5.89×10^6^
153/11	Yellow mongoose	Mongoose variant	1.66×10^7^
177/11	Yellow mongoose	Mongoose variant	6.73×10^3^
448/12	Yellow mongoose	Mongoose variant	2.38×10^7^
502/12	Yellow mongoose	Mongoose variant	2.25×10^7^
594/11	Black-backed jackal	Mongoose variant	4.98×10^6^
1029/99	Bovine	Mongoose variant	4.46×10^4^
1086/99	Bovine	Mongoose variant	7.68×10^4^
CNS samples delivering false-negative results with Monoclonal antibody #2			
306/12	Feline	Mongoose variant	1.15×10^4^
529/99	Yellow mongoose	Mongoose variant	1.60×10^4^
CNS samples delivering false-negative results with both monoclonal antibodies			
1003/99	Canid	Mongoose variant	4.35×10^3^
579/11	Canid	Mongoose variant	2.70×10^6^
133/12	Canid	Mongoose variant	1.39×10^4^
283/11	Feline	Mongoose variant	1.71×10^5^
520/11	Feline	Mongoose variant	2.73×10^4^
613/11	Feline	Mongoose variant	4.01×10^2^
457/12	Feline	Mongoose variant	7.89×10^3^
650/12	Feline	Mongoose variant	9.37×10^5^
651/12	Feline	Mongoose variant	1.25×10^4^
91/11	Yellow mongoose	Mongoose variant	5.00×10^4^
99/11	Yellow mongoose	Mongoose variant	2.87×10^3^
169/11	Yellow mongoose	Mongoose variant	1.31×10^5^
010/12	Yellow mongoose	Mongoose variant	3.13×10^4^
072/12	Yellow mongoose	Mongoose variant	1.50×10^5^
100/12	Yellow mongoose	Mongoose variant	1.63×10^4^
131/12	Yellow mongoose	Mongoose variant	8.22×10^4^
107/12	Bovine	Mongoose variant	2.81×10^3^
CNS samples delivering true-positive results with both Monoclonal antibodies			
1000/99	Yellow mongoose	Mongoose variant	1.17×10^3^
098/11	Yellow mongoose	Mongoose variant	1.98×10^4^
149/11	Yellow mongoose	Mongoose variant	7.96×10^2^
267/11	Yellow mongoose	Mongoose variant	2.85×10^3^
605/11	Yellow mongoose	Mongoose variant	1.30×10^3^
159/12	Yellow mongoose	Mongoose variant	9.42×10^1^
286/12	Yellow mongoose	Mongoose variant	2.30×10^4^

# Canid – *Canis familiaris*; Feline - *Felis domesticus*; Black-backed jackal - *Canis mesomelas*; Yellow mongoose - *Cynictis penicillata*; Bovine - *Bos taurus*.

#### Antigenic typing

The antigenic typing performed in this study indicated that all the isolates in question belonged to a mongoose RABV variant based on the observed immunoreactivity patterns associated with the panel of 16 MAbs (Table 1).

#### Quantification of the viral RNA copy numbers of false negative results

To test a hypothesis that MAb 1 and MAb 2 could have been over-diluted and not optimized for application to the mongoose RABV variant, the viral RNA copy per nanogram of total isolated RNA was calculated (Table 1). Since the viral copy number of samples that tested positive with both MAbs was lower than some of those that tested negative with either one or both of the MAbs, the observed false-negative results were not explained exclusively by viral copy number (Table 1).

### Statistical analysis

The number of true-positive (n = 206) and negative (n = 49) samples was used to perform the statistical analysis of the final results (Table 2). The DFA test had produced a single false-negative result, and had a slightly reduced diagnostic sensitivity of 99.51% (Table 2). The PAb dRIT diagnostic assay had a marginally higher diagnostic efficacy, because all the samples included in the sample set had been diagnosed correctly as either RABV-positive or -negative. As such, the diagnostic sensitivity and specificity of the PAb dRIT was calculated to be 100%, respectively (Table 2).

**Table 2:** Diagnostic sensitivity, specificity and Cohen's Kappa measure of agreement of the direct rapid immunohistochemical test using one of three biotinylated antibody preparations as well as the theoretical monoclonal antibody cocktail evaluated in this study.

DFA							
Biotinylated Antibodies	True Positive	False Positive	True Negative	False Negative	Diagnostic Sensitivity *	Diagnostic Specificity *	Kappa Value *
**Polyclonal Antibody**	205	0	49	1	99.51% (97.31%–99.92%)	100% (92.68%–100%)	—
**dRIT**							
**Polyclonal Antibody**	206	0	49	0	100% (98.21%–100%)	100% (92.68%–100%)	0.987 (0.963–1.000)
**Monoclonal antibody #1**	172	0	49	34	83.50% (77.71%–88.29%)	100% (92.68%–100%)	0,649 (0.548–0,751)
**Monoclonal antibody #2**	187	1	48	19	90.78% (85.97%–94.35%)	97.96% (89.10%–99.66%)	0,767 (0.674–0.861)
**Theoretical Monoclonal antibody cocktail**	189	1	48	17	91.75% (87.11%–95.12%)	97.96% (89.10%–99.66%)	0.832 (0.751–0.914)

*Values in brackets represented the 95% confidence interval (CI).

In contrast to the findings of the pilot dRIT studies released to date [14], [16]–[19], the dRIT diagnostic assay relying on either of the two-biotinylated MAbs had a reduced diagnostic efficacy because of the increased number of incorrect results (Table 2). The dRIT assay using MAb 1 had a decreased diagnostic sensitivity of 83.50% due to the higher number of false-negative results (n = 34), while the dRIT assay using MAb 2 had a diagnostic sensitivity of 90.78% and a slightly reduced diagnostic specificity of 97.96% due to a single false-positive case (Table 2).

The theoretical diagnostic sensitivity and specificity of the dRIT assay using the MAb cocktail was also calculated based on the assumption that false-negative results would only occur when both MAbs produced false-negative results and false-positive results would occur when a single MAb produced a false-positive result (Table 2). The theoretical MAb cocktail thus had a diagnostic sensitivity of 91.75% and a diagnostic specificity of 97.96% (Table 2).

## Discussion

The general applicability of a novel diagnostic assay, in terms of providing a reliable test that can supplement or replace the gold standard DFA test, is primarily determined by the diagnostic sensitivity and specificity of the suggested alternative. Regardless that the diagnostic effectiveness is one of the most important factors, assays must be easy to perform and interpreted in a qualitative manner. In addition, the test should rely on reagents and equipment that are inexpensive and easy to procure and maintain.

Previous published applications of the dRIT [14], [16]–[19] have shown that this assay produced rapid immunoreactivity patterns that were easily observed on a compound light microscope, while maintaining a diagnostic efficacy that is equal to that of the DFA test. One potential hindrance associated with the widespread application of the dRIT diagnostic assay in developing countries may be a lack of a commercial supplier of the biotinylated MAb cocktail. Considering this, our study endeavoured to determine whether it would be straightforward and effective to prepare an alternative biotinylated antibody as replacement for a biotinylated MAb cocktail in the established dRIT diagnostic test.

Thus, an unlabelled PAb, prepared in goats at the ARC-OVI in South Africa, was conjugated to a biotin moiety and the PAb dRIT assay applied to a cohort of samples in parallel with the recommended DFA assay. The results of this study indicated that the PAb dRIT had a diagnostic sensitivity and specificity of 100%, which was marginally higher than that of the DFA. Despite the single false-negative result associated with the DFA test, the diagnostic efficacy of the assay was still within the acceptable limits (98–100%) recommended by the WHO [25]. In contrast, the dRIT using two-biotinylated MAbs was found to be less effective individually in terms of their diagnostic sensitivity once compared to the DFA test under these protocol conditions. Upon calculating the diagnostic efficacy of a theoretical MAb cocktail, the diagnostic sensitivity increased slighly while the diagnostic efficacy remained marginally lower than 100%. Although the diagnostic efficacy of the theoretical MAb cocktail was slightly higher than that of the two individual MAbs, it was lower than the efficacy of the PAb dRIT. Upon closer inspection of the immunoreactivity scores observed in this study, it was clear that PAb dRIT did not only have a superior diagnostic efficacy in comparison to that of the dRIT using the MAbs, but that a higher immunoreactivity scores (+3 and +4) were observed for some samples that had produced a lower immunoreactivity score (+1) once the dRIT relying on either of the MAbs was applied (Table S1). Although this finding did not influence the diagnostic efficacy of the dRIT relying on any of the antibody preparations, the ease with which samples are interpreted is an important point. Samples with a higher immunoreactivity score (+3 and +4) remain easier to diagnose correctly, especially when the test is performed by technicians with limited experience or an ill-equipped laboratory.

The two individual MAbs had a significantly lower diagnostic sensitivity, which was directly attributed to the high number of false-negative results produced by the MAb-based dRIT diagnostic reactions on some, but not all, of the mongoose RABV specimens. The antigenic typing of the RABV variant that produced false-negative results, as well as the remaining mongoose samples that were correctly diagnosed as RABV-positive by both MAbs, were all found to be associated with the mongoose variant of the RABV. To our knowledge, this was the first application of the dRIT assay using either of the two-biotinylated MAbs (MAb 1 or MAb 2) to the mongoose RABV variant. The inclusion of an African mongoose RABV variant thus resulted in the first demonstration whereby the classical dRIT has shown a reduced diagnostic efficacy. However, the two MAbs used in the study were supplied as “ready-to-use” solutions, and as such the working dilutions could not be optimized on the sample cohort included in this study. To investigate that the working dilution of each of the MAbs might have been over-diluted, a quantitative real-time PCR analysis of the RABV RNA concentration in each of the samples was performed. Although not indicative of the number of assembled RABV particles, the RNA concentration was used to determine whether a cut-off value could be determined whereby the inconsistency of the dRIT assay using the MAbs could be explained. Because no clear cut-off values in terms of the viral RNA copy number could be determined between the specimens that produced true-positive and false-negative results, the hypothesis that the MAbs had been over-diluted prior to shipment could not be easily addressed adequately. We also argue that it is not copy number alone that influences avidity, but the relative availability of antigenic sites recognized by a given MAb based upon variant epitopes, largely determined based on the panel of viruses used for the optimization of the assay. For the MAbs used here, the majority of the test panel had been primarily New World wildlife variants, including viruses isolated from mongoose (Caribbean), which are unlike mongoose viruses from southern Africa. With this in mind, any future application of the dRIT assay, using any biotinylated antibody preparations, should be preceded by the optimization of the antibody preparation for the given assay based upon variants of public health importance to the region. Clearly, this was an unequal advantage in the protocol evaluated here, because the MAb preparations were pre-diluted based upon other RABV variants whereas the PAb was optimized based upon local production.

Another potential explanation for the observed false negative results could be that the two-biotinylated MAb preparations interact with single epitopes on antigenic sites that are less conserved in certain mongoose variants present in sub-Saharan Africa. The two-MAbs used in this study each interact with a different epitope on the nucleoprotein. A single change at the nucleotide level could theoretically result in a change in the translated amino acid sequence of the virus, resulting in altered epitopes with which the MAbs cannot interact. Despite the obvious advantages that MAb preparations have in terms of the lot-to-lot consistency, sustainable supply from immortal cell lines without the further use of animals and comparatively inexpensive costs [33], [34], PAb preparations have the benefit of associating with multiple epitopes on various antigenic sites [33], [34]. This advantageous trait enables diagnostic reactions to be influenced to a potentially lesser degree by the high rate of mutation observed in lyssaviruses.

The application of the dRIT diagnostic reaction produced no false-negative results when applied to the six representative southern African rabies-related isolates. Although not all of the currently known African lyssavirus isolates were included in this study, the successful diagnosis of the representative isolates was used as an initial proof of concept to highlight the utility of the dRIT diagnostic assay as applied to antigenically and genetically distinct lyssavirus species.

Although numerous novel diagnostic assays, such as molecular amplification and lateral flow immunochromatographic assays (rapid test kits), are being developed and may show promise in terms of becoming viable options for supplementing the DFA test, they are still under development. Such diagnostic tools might have potential future applications, and the widespread implementation of the highly reliable diagnostic assays that are currently available should be encouraged. In this study, the dRIT diagnostic assay has been shown to be one such option. The dRIT test, using the biotinylated PAb preparation, has a diagnostic sensitivity and specificity that is marginally higher than that of the DFA. This fact thus justifies further evaluation of the dRIT diagnostic assay on a global scale to ensure the widespread application of this diagnostic assay, corroborate our preliminary findings and continue to compare the utility of MAbs in the dRIT when antibody concentration and cocktail optimization is not a limiting factor to experimental design.

## Supporting Information

Checklist S1 **STARD checklist.**
(DOC)

Table S1 **Sample details and relevant immunoreactivity scores associated with the direct fluorescent antibody test and direct rapid immunohistochemical test as performed on various southern African maintenance hosts.**
(DOCX)

Table S2 **Sample details and relevant immunoreactivity scores associated with the direct fluorescent antibody test and direct rapid immunohistochemical test as performed on six representative rabies-related lyssaviruses from southern Africa.**
(DOCX)
