# Supplementary material for: Comparison of Biotinylated Monoclonal and Polyclonal Antibodies in an Evaluation of a Direct Rapid Immunohistochemical Test for the Routine Diagnosis of Rabies in Southern Africa
Source: PLoS Negl Trop Dis. 2014 Sep 25;8(9):e3189. doi: 10.1371/journal.pntd.0003189 (PMC4177867; doi:10.1371/journal.pntd.0003189)
Supplement: Checklist S1 — STARD checklist. (DOC) [file pntd.0003189.s001.doc]

**STARD checklist for the study: “Comparison of biotinylated monoclonal and polyclonal antibodies in an evaluation of a direct rapid immunohistochemical test for the routine diagnosis of rabies in southern Africa”, submitted at PLoS NTDs**

**Item 1: Identify the article as a study of diagnostic accuracy.**

An open title was chosen for this paper.

**Item 2: State the research questions or study aims, such as estimating diagnostic accuracy or comparing accuracy between tests or across participant groups.**

The aim(s) of this study were clearly stated on page #5.

“The objective of this study was to test the hypothesis that an alternative polyclonal antibody (PAb) preparation could be biotinylated and applied in the dRIT diagnostic reaction with equal or improved results compared to the use of the MAbs.”

**Item 3: The study population: The inclusion and exclusion criteria, setting and locations where data were collected.**

Study population: Central nervous system tissues submitted to the ARC-OVI, Rabies Division, for routine rabies diagnosis were used in this study. The samples had been diagnosed for notification purposes and were used in this study retrospectively. The study population is discussed in more detail under the heading “sample size and selection” on page #6.

**Item 4: Participant recruitment: Was recruitment based on presenting symptoms, results from previous tests, or the fact that the participants had received the index tests or the reference standard?**

Animal rabies is a notifiable disease in South Africa. State veterinarians had submitted samples to the ARC-OVI, Rabies Division, for routine rabies diagnosis based on suspicion of rabies. The participant recruitment is discussed in more detail under the heading “sample size and selection” on page #6.

**Item 5: Participant sampling: Was the study population a consecutive series of participants defined by the selection criteria in item 3 and 4? If not, specify how participants were further selected.**

No, samples were chosen based on their importance as maintenance host species collected over three annual time periods. Dog samples were given preference and an equal number of samples for the remaining species were chosen. The participant sampling is discussed in more detail under the heading “sample size and selection” on page #6.

**Item 6: Data collection: Was data collection planned before the index test and reference standard were performed (prospective study) or after (retrospective study)?**

The data collection was planned before the performance of the index test and reference test.

Samples had been tested with the DFA test on arrival at the ARC-OVI, Rabies Division, for routine rabies diagnosis. Both the DFA and DRIT assays were performed retrospectively on each of the samples during the course of this study. The data collection is discussed in more detail under the heading “sample size and selection” on page #6.

**Item 7: The reference standard and its rationale.**

The reference standard test is the DFA (direct fluorescent antibody) test. This is the diagnostic assay recommended by both the WHO and OIE and is considered the “gold standard” diagnostic assay for diagnosis of rabies virus based on the presence of viral antigen. The reference test is discussed in more detail and cited under the heading “direct fluorescent antibody test” on page #7.

**Item 8: Technical specifications of material and methods involved including how and when measurements were taken, and/or cite references for index tests and reference standard.**

The standard operating procedure for the index test (DRIT), as well as an up-to-date publication list of all the studies relying on the assay, is mentioned and cited on page #8. The index test is discussed in more detail and cited under the heading “direct rapid immunohistochemical test” on page #8.

**Item 9: Definition of and rationale for the units, cut-offs and/or categories of the results of the index tests and the reference standard.**

The guidelines used to discern the immunoreactivity scores associated with all the samples are discussed in the footer of Table S1 in the supplementary material.

**Item 10: The number, training and expertise of the persons executing and reading the index tests and the reference standard.**

The initial DFA assay was performed by two trained diagnostic technicians from the ARC-OVI, Rabies Division. The second round of DFA performed on each samples was done by a researcher from the University of Pretoria (Author: Andre Coetzer) who had undergone training from the aforementioned Rabies Division staff. The DRIT assay was performed by a researcher from the University of Pretoria (Author: Andre Coetzer).

**Item 11: Whether or not the readers of the index tests and reference standard were blind (masked) to the results of the other test and describe any other clinical information available to the readers.**

The reading of the reference and index tests were done blindly. The DFA result was thus unknown when the DRIT results were interpreted.

**Item 12: Methods for calculating or comparing measures of diagnostic accuracy, and the statistical methods used to quantify uncertainty (e.g. 95% confidence intervals).**

The statistical analysis of the diagnostic efficacy of the DRIT test was based on the formulas for determining diagnostic sensitivity, specificity and the Cohen Kappa measure of agreement. The statistical analysis is discussed in more detail under the heading “data analysis” on page #10.

**Item 13: Methods for calculating test reproducibility, if done.**

For any samples that produced a different result with any of the test, all the tests were repeated a further 3 times. There were no instances of changed results after the repetition.

**Item 14: When study was performed, including beginning and end dates of recruitment.**

The work presented in this study was performed between 16 March 2012 and 26 October 2012. The samples had been submitted to the ARC-OVI, rabies division, over three different annual periods mentioned in the “sample size and selection” heading on page #6.

**Item 15: Clinical and demographic characteristics of the study population (at least information on age, gender, spectrum of presenting symptoms).**

Sample information summarized in supplementary material – Table S1 and S2. Further information can be obtained from the corresponding author.

**Item 16: The number of participants satisfying the criteria for inclusion who did or did not undergo the index tests and/or the reference standard; describe why participants failed to undergo either test (a flow diagram is strongly recommended).**

No such cases found in this study.

**Item 17: Time-interval between the index tests and the reference standard, and any treatment administered in between.**

Samples were split into smaller groups and the DFA and DRIT were performed on two consecutive days (DFA and DRIT were performed approximately 24 hours apart.)

**Item 18: Distribution of severity of disease (define criteria) in those with the target condition; other diagnoses in participants without the target condition.**

Not applicable

**Item 19: A cross tabulation of the results of the index tests (including indeterminate and missing results) by the results of the reference standard; for continuous results, the distribution of the test results by the results of the reference standard.**

See Table 2 on page #21.

**Item 20: Any adverse events from performing the index tests or the reference standard.**

Not applicable

**Item 21: Estimates of diagnostic accuracy and measures of statistical uncertainty (e.g. 95% confidence intervals).**

See Table 2 on page #21.

**Item 22: How indeterminate results, missing data and outliers of the index tests were handled.**

Not applicable

**Item 23: Estimates of variability of diagnostic accuracy between subgroups of participants, readers or centers, if done.**

Not done in this study

**Item 24: Estimates of test reproducibility, if done.**

All the DFA positive samples were tested positive with the PAb DRIT relying on blind testing.

**Item 25: Discuss the clinical applicability of the study findings.**

The applicability of the study is discussed in more detail under the heading “conclusion” on page #13.
