# Supplementary material for: Comparison of Biotinylated Monoclonal and Polyclonal Antibodies in an Evaluation of a Direct Rapid Immunohistochemical Test for the Routine Diagnosis of Rabies in Southern Africa
Source: PLoS Negl Trop Dis. 2014 Sep 25;8(9):e3189. doi: 10.1371/journal.pntd.0003189 (PMC4177867; doi:10.1371/journal.pntd.0003189)
Supplement: Table S1 — Sample details and relevant immunoreactivity scores associated with the direct fluorescent antibody test and direct rapid immunohistochemical test as performed on various southern African maintenance hosts. (DOCX) [file pntd.0003189.s002.docx]

**Table S1. Sample details and relevant immunoreactivity scores associated with the direct fluorescent antibody test and direct rapid immunohistochemical test as performed on various southern African maintenance hosts.**

| **Sample information** | | | **FAT** | **dRIT** | | |
| --- | --- | --- | --- | --- | --- | --- |
| **Sample Number** | **Year** | **Animal^#^** |  | **MAb 1** | **MAb 2** | **PAb** |
| 208/99 | 1999 | CAN | ++ | + | ++ | ++ |
| 273/99 | 1999 | CAN | +++ | ++ | ++ | +++ |
| 524/99 | 1999 | CAN | ++ | + | + | ++ |
| 596/99 | 1999 | CAN | ++ | +++ | +++ | + |
| 687/99 | 1999 | CAN | +++ | +++ | +++ | + |
| 756/99 | 1999 | CAN | +++ | - - - | ++ | + |
| 828/99 | 1999 | CAN | +++ | + | + | ++++ |
| 1003/99 | 1999 | CAN | ++ | - - - | - - - | + |
| 1018/99 | 1999 | CAN | +++ | + | + | ++ |
| 1039/99 | 1999 | CAN | ++ | + | + | ++ |
| 007/11 | 2011 | CAN | ++++ | ++++ | +++ | ++++ |
| 035/11 | 2011 | CAN | ++++ | ++++ | ++++ | ++++ |
| 042/11 | 2011 | CAN | +++ | ++++ | ++++ | ++++ |
| 045/11 | 2011 | CAN | ++++ | ++++ | ++++ | ++++ |
| 074/11 | 2011 | CAN | +++ | +++ | +++ | +++ |
| 104/11 | 2011 | CAN | +++ | +++ | +++ | +++ |
| 106/11 | 2011 | CAN | ++++ | ++++ | ++++ | ++++ |
| 107/11 | 2011 | CAN | ++++ | ++++ | ++++ | ++++ |
| 108/11 | 2011 | CAN | ++++ | ++++ | ++++ | ++++ |
| 109/11 | 2011 | CAN | +++ | +++ | +++ | +++ |
| 127/11 | 2011 | CAN | +++ | ++++ | ++++ | ++++ |
| 131/11 | 2011 | CAN | +++ | +++ | +++ | +++ |
| 132/11 | 2011 | CAN | +++ | +++ | ++++ | +++ |
| 139/11 | 2011 | CAN | ++++ | +++ | ++++ | ++++ |
| 154/11 | 2011 | CAN | ++++ | ++++ | ++++ | ++++ |
| 167/11 | 2011 | CAN | ++++ | ++++ | ++++ | ++++ |
| 168/11 | 2011 | CAN | ++++ | ++++ | ++++ | ++++ |
| 172/11 | 2011 | CAN | +++ | +++ | +++ | ++++ |
| 187/11 | 2011 | CAN | +++ | +++ | +++ | +++ |
| 206/11 | 2011 | CAN | +++ | ++++ | ++++ | ++++ |
| 212/11 | 2011 | CAN | ++ | +++ | +++ | +++ |
| 247/11 | 2011 | CAN | +++ | +++ | +++ | +++ |
| 264/11 | 2011 | CAN | ++ | +++ | +++ | +++ |
| 274/11 | 2011 | CAN | ++++ | ++++ | ++++ | ++++ |
| 276/11 | 2011 | CAN | ++++ | ++++ | ++++ | ++++ |
| 285/11 | 2011 | CAN | +++ | +++ | +++ | +++ |
| 312/11 | 2011 | CAN | +++ | +++ | +++ | ++++ |
| 315/11 | 2011 | CAN | ++++ | ++++ | ++++ | ++++ |
| 395/11 | 2011 | CAN | + | ++ | ++ | ++ |
| 401/11 | 2011 | CAN | ++++ | ++++ | ++++ | ++++ |
| 418/11 | 2011 | CAN | ++++ | ++++ | +++ | ++++ |
| 424/11 | 2011 | CAN | +++ | +++ | +++ | +++ |
| 434/11 | 2011 | CAN | ++ | ++ | + | ++ |
| 436/11 | 2011 | CAN | +++ | +++ | +++ | +++ |
| 443/11 | 2011 | CAN | +++ | ++++ | ++++ | ++++ |
| 446/11 | 2011 | CAN | +++ | ++++ | ++++ | ++++ |
| 455/11 | 2011 | CAN | ++ | ++ | ++ | ++ |
| 487/11 | 2011 | CAN | +++ | ++++ | ++++ | ++++ |
| 502/11 | 2011 | CAN | +++ | ++++ | ++++ | ++++ |
| 512/11 | 2011 | CAN | + | + | + | + |
| 515/11 | 2011 | CAN | ++++ | ++++ | ++++ | ++++ |
| 526/11 | 2011 | CAN | ++ | ++ | ++ | ++ |
| 528/11 | 2011 | CAN | +++ | +++ | +++ | +++ |
| 579/11 | 2011 | CAN | ++++ | - - - | - - - | +++ |
| 590/11 | 2011 | CAN | ++++ | ++++ | ++++ | ++++ |
| 596/11 | 2011 | CAN | +++ | +++ | +++ | +++ |
| 618/11 | 2011 | CAN | ++++ | ++++ | ++++ | ++++ |
| 627/11 | 2011 | CAN | ++ | ++ | ++ | ++ |
| 634/11 | 2011 | CAN | ++ | +++ | +++ | +++ |
| 644/11 | 2011 | CAN | +++ | ++ | ++ | ++ |
| 646/11 | 2011 | CAN | +++ | ++++ | ++++ | ++++ |
| 665/11 | 2011 | CAN | ++++ | ++++ | ++++ | ++++ |
| 681/11 | 2011 | CAN | ++++ | ++++ | ++++ | ++++ |
| 685/11 | 2011 | CAN | ++ | ++ | ++ | ++ |
| 686/11 | 2011 | CAN | +++ | ++++ | ++++ | ++++ |
| 708/11 | 2011 | CAN | +++ | +++ | ++ | +++ |
| 726/11 | 2011 | CAN | ++++ | ++++ | ++++ | ++++ |
| 738/11 | 2011 | CAN | ++++ | +++ | ++ | +++ |
| 758/11 | 2011 | CAN | ++ | +++ | ++ | +++ |
| 777/11 | 2011 | CAN | ++++ | ++++ | ++++ | ++++ |
| 786/11 | 2011 | CAN | ++++ | +++ | +++ | +++ |
| 801/11 | 2011 | CAN | ++++ | +++ | +++ | +++ |
| 833/11 | 2011 | CAN | +++ | ++++ | +++ | +++ |
| 837/11 | 2011 | CAN | + | + | + | + |
| 840/11 | 2011 | CAN | +++ | ++++ | ++++ | ++++ |
| 843/11 | 2011 | CAN | + | ++ | +++ | ++ |
| 862/11 | 2011 | CAN | ++ | ++ | ++ | ++ |
| 869/11 | 2011 | CAN | ++++ | ++++ | ++++ | ++++ |
| 884/11 | 2011 | CAN | +++ | +++ | ++ | +++ |
| 889/11 | 2011 | CAN | ++++ | ++++ | ++++ | ++++ |
| 019/12 | 2012 | CAN | ++++ | ++++ | ++++ | ++++ |
| 037/12 | 2012 | CAN | ++++ | ++++ | ++++ | ++++ |
| 049/12 | 2012 | CAN | ++++ | ++++ | ++++ | ++++ |
| 077/12 | 2012 | CAN | +++ | +++ | +++ | +++ |
| 097/12 | 2012 | CAN | ++++ | ++++ | ++++ | ++++ |
| 133/12 | 2012 | CAN | +++ | - - - | - - - | ++++ |
| 136/12 | 2012 | CAN | ++++ | ++++ | ++++ | ++++ |
| 172/12 | 2012 | CAN | ++++ | ++++ | ++++ | ++++ |
| 185/12 | 2012 | CAN | ++ | ++ | ++ | ++ |
| 200/12 | 2012 | CAN | +++ | +++ | +++ | +++ |
| 229/12 | 2012 | CAN | ++++ | ++++ | ++++ | ++++ |
| 235/12 | 2012 | CAN | ++++ | ++++ | ++++ | ++++ |
| 274/12 | 2012 | CAN | +++ | ++++ | +++ | ++++ |
| 319/12 | 2012 | CAN | ++++ | ++++ | ++++ | ++++ |
| 324/12 | 2012 | CAN | +++ | +++ | +++ | +++ |
| 359/12 | 2012 | CAN | ++++ | ++ | ++ | ++++ |
| 371/12 | 2012 | CAN | ++++ | +++ | ++++ | ++++ |
| 400/12 | 2012 | CAN | ++++ | ++++ | ++++ | ++++ |
| 423/12 | 2012 | CAN | ++++ | ++++ | ++++ | ++++ |
| 458/12 | 2012 | CAN | ++++ | ++++ | ++++ | ++++ |
| 468/12 | 2012 | CAN | +++ | +++ | +++ | +++ |
| 508/12 | 2012 | CAN | ++++ | ++++ | ++++ | ++++ |
| 711/12 | 2012 | CAN | - - - | + | + | + |
| 664/12 | 2012 | CAN | - - - | - - - | + | - - - |
| 665/12 | 2012 | CAN | - - - | - - - | - - - | - - - |
| 669/12 | 2012 | CAN | - - - | - - - | - - - | - - - |
| 672/12 | 2012 | CAN | - - - | - - - | - - - | - - - |
| 675/12 | 2012 | CAN | - - - | - - - | - - - | - - - |
| 678/12 | 2012 | CAN | - - - | - - - | - - - | - - - |
| 679/12 | 2012 | CAN | - - - | - - - | - - - | - - - |
| 680/12 | 2012 | CAN | - - - | - - - | - - - | - - - |
| 684/12 | 2012 | CAN | - - - | - - - | - - - | - - - |
| 687/12 | 2012 | CAN | - - - | - - - | - - - | - - - |
| 688/12 | 2012 | CAN | - - - | - - - | - - - | - - - |
| 697/12 | 2012 | CAN | - - - | - - - | - - - | - - - |
| 701/12 | 2012 | CAN | - - - | - - - | - - - | - - - |
| 702/12 | 2012 | CAN | - - - | - - - | - - - | - - - |
| 707/12 | 2012 | CAN | - - - | - - - | - - - | - - - |
| 710/12 | 2012 | CAN | - - - | - - - | - - - | - - - |
| 715/12 | 2012 | CAN | - - - | - - - | - - - | - - - |
| 720/12 | 2012 | CAN | - - - | - - - | - - - | - - - |
| 740/12 | 2012 | CAN | - - - | - - - | - - - | - - - |
| 743/12 | 2012 | CAN | - - - | - - - | - - - | - - - |
| 745/12 | 2012 | CAN | - - - | - - - | - - - | - - - |
| 748/12 | 2012 | CAN | - - - | - - - | - - - | - - - |
| 756/12 | 2012 | CAN | - - - | - - - | - - - | - - - |
| 765/12 | 2012 | CAN | - - - | - - - | - - - | - - - |
| 766/12 | 2012 | CAN | - - - | - - - | - - - | - - - |
| 769/12 | 2012 | CAN | - - - | - - - | - - - | - - - |
| 773/12 | 2012 | CAN | - - - | - - - | - - - | - - - |
| 775/12 | 2012 | CAN | - - - | - - - | - - - | - - - |
| 777/12 | 2012 | CAN | - - - | - - - | - - - | - - - |
| 601/99 | 1999 | FEL | ++++ | - - - | ++++ | ++++ |
| 620/99 | 1999 | FEL | ++++ | - - - | + | +++ |
| 929/99 | 1999 | FEL | +++ | + | + | + |
| 1052/99 | 1999 | FEL | ++++ | +++ | +++ | +++ |
| 114/11 | 2011 | FEL | ++ | - - - | + | +++ |
| 283/11 | 2011 | FEL | ++++ | - - - | - - - | ++++ |
| 376/11 | 2011 | FEL | ++ | - - - | + | +++ |
| 467/11 | 2011 | FEL | ++ | +++ | + | +++ |
| 481/11 | 2011 | FEL | ++++ | ++++ | ++++ | ++++ |
| 520/11 | 2011 | FEL | ++ | - - - | - - - | ++++ |
| 613/11 | 2011 | FEL | +++ | - - - | - - - | +++ |
| 650/11 | 2011 | FEL | + | ++ | ++ | ++ |
| 660/11 | 2011 | FEL | +++ | - - - | + | ++++ |
| 846/11 | 2011 | FEL | +++ | + | + | +++ |
| 051/12 | 2012 | FEL | +++ | +++ | +++ | +++ |
| 261/12 | 2012 | FEL | ++++ | - - - | ++++ | ++++ |
| 306/12 | 2012 | FEL | +++ | + | - - - | +++ |
| 345/12 | 2012 | FEL | + | + | + | ++ |
| 382/12 | 2012 | FEL | ++++ | - - - | +++ | +++ |
| 457/12 | 2012 | FEL | ++ | - - - | - - - | +++ |
| 650/12 | 2012 | FEL | ++++ | - - - | - - - | ++++ |
| 651/12 | 2012 | FEL | +++ | - - - | - - - | +++ |
| 391/12 | 2012 | FEL | - - - | - - - | - - - | - - - |
| 414/12 | 2012 | FEL | - - - | - - - | - - - | - - - |
| 432/12 | 2012 | FEL | - - - | - - - | - - - | - - - |
| 443/12 | 2012 | FEL | - - - | - - - | - - - | - - - |
| 744/12 | 2012 | FEL | - - - | - - - | - - - | - - - |
| 529/99 | 1999 | CPEN | ++++ | + | - - - | ++++ |
| 540/99 | 1999 | CPEN | +++ | - - - | +++ | +++ |
| 1000/99 | 1999 | CPEN | + | + | + | + |
| 1087/99 | 1999 | CPEN | +++ | - - - | + | +++ |
| 091/11 | 2011 | CPEN | ++++ | - - - | - - - | ++++ |
| 098/11 | 2011 | CPEN | ++++ | +++ | +++ | ++++ |
| 099/11 | 2011 | CPEN | ++++ | - - - | - - - | ++++ |
| 149/11 | 2011 | CPEN | + | + | + | + |
| 153/11 | 2011 | CPEN | +++ | - - - | ++++ | ++++ |
| 169/11 | 2011 | CPEN | ++++ | - - - | - - - | ++++ |
| 177/11 | 2011 | CPEN | + | - - - | + | +++ |
| 267/11 | 2011 | CPEN | ++++ | + | + | ++++ |
| 605/11 | 2011 | CPEN | + | + | + | ++ |
| 010/12 | 2012 | CPEN | +++ | - - - | - - - | +++ |
| 072/12 | 2012 | CPEN | ++++ | - - - | - - - | ++++ |
| 100/12 | 2012 | CPEN | ++++ | - - - | - - - | ++++ |
| 131/12 | 2012 | CPEN | ++++ | - - - | - - - | ++++ |
| 159/12 | 2012 | CPEN | + | + | + | + |
| 268/12 | 2012 | CPEN | +++ | + | + | ++++ |
| 448/12 | 2012 | CPEN | ++++ | - - - | +++ | ++++ |
| 502/12 | 2012 | CPEN | ++++ | - - - | ++++ | ++++ |
| 401/12 | 2012 | CPEN | - - - | - - - | - - - | - - - |
| 405/12 | 2012 | CPEN | - - - | - - - | - - - | - - - |
| 441/12 | 2012 | CPEN | - - - | - - - | - - - | - - - |
| 751/12 | 2012 | CPEN | - - - | - - - | - - - | - - - |
| 755/12 | 2012 | CPEN | - - - | - - - | - - - | - - - |
| 557/99 | 1999 | CMES | +++ | ++ | +++ | ++++ |
| 549/99 | 1999 | CMES | +++ | +++ | ++++ | ++++ |
| 673/99 | 1999 | CMES | ++ | +++ | +++ | ++++ |
| 717/99 | 1999 | CMES | ++++ | +++ | +++ | ++++ |
| 121/11 | 2011 | CMES | ++++ | +++ | +++ | ++++ |
| 138/11 | 2011 | CMES | +++ | +++ | +++ | +++ |
| 147/11 | 2011 | CMES | ++++ | ++++ | +++ | ++++ |
| 257/11 | 2011 | CMES | ++++ | +++ | ++++ | ++++ |
| 364/11 | 2011 | CMES | ++++ | + | + | ++++ |
| 448/11 | 2011 | CMES | +++ | ++++ | +++ | ++++ |
| 493/11 | 2011 | CMES | ++++ | ++++ | ++++ | ++++ |
| 594/11 | 2011 | CMES | +++ | - - - | + | +++ |
| 633/11 | 2011 | CMES | ++++ | ++++ | ++++ | ++++ |
| 678/11 | 2011 | CMES | + | + | + | + |
| 103/12 | 2012 | CMES | ++ | ++ | ++ | ++ |
| 169/12 | 2012 | CMES | ++++ | ++++ | ++++ | ++++ |
| 249/12 | 2012 | CMES | +++ | +++ | +++ | +++ |
| 266/12 | 2012 | CMES | +++ | ++++ | ++++ | ++++ |
| 433/12 | 2012 | CMES | +++ | ++++ | ++++ | ++++ |
| 494/12 | 2012 | CMES | ++++ | ++++ | ++++ | ++++ |
| 566/12 | 2012 | CMES | ++ | +++ | +++ | +++ |
| 594/12 | 2012 | CMES | ++++ | + | + | ++++ |
| 514/12 | 2012 | CMES | - - - | - - - | - - - | - - - |
| 553/12 | 2012 | CMES | - - - | - - - | - - - | - - - |
| 582/12 | 2012 | CMES | - - - | - - - | - - - | - - - |
| 591/12 | 2012 | CMES | - - - | - - - | - - - | - - - |
| 238/99 | 1999 | OMEG | + | ++ | + | ++ |
| 395/99 | 1999 | OMEG | +++ | ++ | +++ | +++ |
| 971/99 | 1999 | OMEG | +++ | ++++ | +++ | +++ |
| 1082/99 | 1999 | OMEG | +++ | ++ | ++ | +++ |
| 656/11 | 2011 | OMEG | ++ | ++++ | ++++ | +++ |
| 909/11 | 2011 | OMEG | +++ | +++ | + | +++ |
| 095/12 | 2012 | OMEG | +++ | ++++ | ++++ | ++++ |
| 227/12 | 2012 | OMEG | ++++ | ++++ | ++++ | ++++ |
| 313/12 | 2012 | OMEG | +++ | +++ | +++ | +++ |
| 642/12 | 2012 | OMEG | +++ | +++ | +++ | +++ |
| 758/12 | 2012 | OMEG | - - - | - - - | - - - | - - - |
| 388/99 | 1999 | BOV | +++ | +++ | +++ | +++ |
| 406/99 | 1999 | BOV | ++++ | + | + | ++++ |
| 1029/99 | 1999 | BOV | ++++ | - - - | + | ++++ |
| 1086/99 | 1999 | BOV | ++++ | - - - | ++ | ++++ |
| 119/11 | 2011 | BOV | ++++ | ++++ | ++++ | ++++ |
| 129/11 | 2011 | BOV | ++++ | ++++ | ++++ | ++++ |
| 279/11 | 2011 | BOV | ++++ | ++++ | ++++ | ++++ |
| 302/11 | 2011 | BOV | ++ | ++ | ++ | ++ |
| 313/11 | 2011 | BOV | ++++ | +++ | ++++ | ++++ |
| 344/11 | 2011 | BOV | ++++ | + | + | ++++ |
| 472/11 | 2011 | BOV | ++ | ++ | + | ++ |
| 479/11 | 2011 | BOV | +++ | +++ | +++ | +++ |
| 879/11 | 2011 | BOV | ++++ | ++++ | ++++ | ++++ |
| 885/11 | 2011 | BOV | +++ | ++++ | ++++ | ++++ |
| 011/12 | 2012 | BOV | ++++ | ++++ | ++++ | ++++ |
| 030/12 | 2012 | BOV | +++ | ++++ | ++++ | ++++ |
| 071/12 | 2012 | BOV | ++ | +++ | +++ | +++ |
| 107/12 | 2012 | BOV | ++++ | - - - | - - - | ++++ |
| 201/12 | 2012 | BOV | ++ | ++ | ++ | ++ |
| 331/12 | 2012 | BOV | ++++ | ++++ | ++++ | ++++ |
| 412/12 | 2012 | BOV | ++++ | ++++ | ++++ | ++++ |
| 490/12 | 2012 | BOV | ++++ | + | + | ++++ |
| 409/12 | 2012 | BOV | - - - | - - - | - - - | - - - |
| 445/12 | 2012 | BOV | - - - | - - - | - - - | - - - |
| 745/12 | 2012 | BOV | - - - | - - - | - - - | - - - |
| 767/12 | 2012 | BOV | - - - | - - - | - - - | - - - |
| 776/12 | 2012 | BOV | - - - | - - - | - - - | - - - |
| # Can – Canid (*Canis familiaris*); Fel – Feline (*Felis domesticus*); CPEN – Yellow mongoose (*Cynictis penicillata*); CMES – Black-backed jackal (*Canis mesomelas*); OMEG – Bat-eared fox (*Otocyon megalotis*); Bov – Bovine (*Bos taurus*)  - - -: No particles present in any of the fields. Sample is considered to be negative  +: The antigen is very scanty with one or more particles in less than 50% of the fields  ++: The antigen is scanty with one or more particle present in less than 100%, but in more than 50%, of the fields  +++: The antigen is abundant with one or more particle present in every field, but the amount of antigen present can be counted properly  ++++: The antigen is very abundant in every field and the amount of antigen present is “too numerous to count properly” | | | | | | |
