# Supplementary material for: Comparison of Biotinylated Monoclonal and Polyclonal Antibodies in an Evaluation of a Direct Rapid Immunohistochemical Test for the Routine Diagnosis of Rabies in Southern Africa
Source: PLoS Negl Trop Dis. 2014 Sep 25;8(9):e3189. doi: 10.1371/journal.pntd.0003189 (PMC4177867; doi:10.1371/journal.pntd.0003189)
Supplement: Table S2 — Sample details and relevant immunoreactivity scores associated with the direct fluorescent antibody test and direct rapid immunohistochemical test as performed on six representative rabies-related lyssaviruses from southern Africa. (DOCX) [file pntd.0003189.s003.docx]

**Table S2. Sample details and relevant immunoreactivity scores associated with the direct fluorescent antibody test and direct rapid immunohistochemical test as performed on six representative rabies-related lyssaviruses from southern Africa**

| **Sample information** | | | | **FAT** | **dRIT** | | |
| --- | --- | --- | --- | --- | --- | --- | --- |
| **Rabies-related lyssavirus** ***** | **Lab reference number** | **Host species** | **Accession nr.** |  | **MAb 1** | **MAb 2** | **PAb** |
| Lagos bat virus (Lineage C) | LBVSA 04 | Bat | DQ499945 | ++++ | ++++ | ++++ | ++++ |
| Mokola virus (Group 1) | 13270/82 | Feline | AF319514 | ++++ | ++++ | ++++ | ++++ |
| Mokola virus (Group 2) | 322/96 | Feline | AF074813 | ++++ | ++++ | ++++ | ++++ |
| Mokola virus (Group 3) | 252/97 | Feline | AF074816 | ++++ | ++++ | ++++ | ++++ |
| Mokola virus (Group 4) | 112/96 | Feline | AF074810 | ++++ | ++++ | ++++ | ++++ |
| Duvenhage virus | DUVVSA 06 | Human | DQ676932 | ++++ | ++++ | ++++ | ++++ |
| ++++: The antigen is very abundant in every field and the amount of antigen present is “too numerous to count properly” | | | | | | | |
